# Altered Temporal Structure of Neural Phase Synchrony in Patients With Autism Spectrum Disorder

**DOI:** 10.3389/fpsyt.2021.618573

**Published:** 2021-11-10

**Authors:** Huibin Jia, Fei Gao, Dongchuan Yu

**Affiliations:** ^1^Institute of Cognition, Brain and Health, Henan University, Kaifeng, China; ^2^School of Psychology, Henan University, Kaifeng, China; ^3^Institute of Psychology and Behavior, Henan University, Kaifeng, China; ^4^Key Laboratory of Child Development and Learning Science of Ministry of Education, Research Center for Learning Science, School of Biological Sciences and Medical Engineering, Southeast University, Nanjing, China; ^5^Department of Pain Medicine, Peking University People's Hospital, Beijing, China

**Keywords:** phase synchrony, long-range temporal correlations, detrended fluctuation analysis, autism spectrum disorder, default mode network, mirror neuron system

## Abstract

Functional connectivity, quantified by phase synchrony, between brain regions is known to be aberrant in patients with autism spectrum disorder (ASD). Here, we evaluated the long-range temporal correlations of time-varying phase synchrony (TV-PS) of electrocortical oscillations in patients with ASD as well as typically developing people using detrended fluctuation analysis (DFA) after validating the scale-invariance of the TV-PS time series. By comparing the DFA exponents between the two groups, we found that those of the TV-PS time series of high-gamma oscillations were significantly attenuated in patients with ASD. Furthermore, the regions involved in aberrant TV-PS time series were mainly within the social ability and cognition-related cortical networks. These results support the notion that abnormal social functions observed in patients with ASD may be caused by the highly volatile phase synchrony states of electrocortical oscillations.

## Introduction

Autism spectrum disorder (ASD) is a group of neurodevelopmental disorders with sociocommunicative impairments, restricted and repetitive behaviors and interests as core diagnostic features (1). Using modern neuroimaging techniques, scientists found that the symptomatology of this disease was not caused by specific cortical regions, but was associated with the hyper- or hypo-connectivity between cortical regions (2, 3). In most published ASD studies, researchers investigated the undirected or directed functional connectivity (FC) between brain regions using measures such as correlation, phase synchrony, and Granger causality, and assumed that these measures were stationary over time (4). However, recent studies have confirmed that FC metrics exhibit variation over time, even during the task-free resting state (5). Moreover, temporal variation in FC metrics can be modulated by various factors, such as brain disorders (6). Surprisingly, the majority of previous studies concentrated on the altered magnitude of functional connectivity between brain regions; few studies have investigated the temporal structure of the time-varying connectivity in the autistic brain.

Long-range temporal correlation (LRTC) is a well-established property of temporal structure in many levels of nervous signals. This property suggests that cortical oscillations have scale-free structures over multiple time scales and work near the critical state (7). This phenomenon offers certain functional benefits for the brain system, such as balanced propagation of external/internal perturbations and maximization of information storage and transfer (8). Detrended fluctuation analysis (DFA) is commonly conducted to quantify LRTCs by amplitude modulation of cortical oscillations (9). For human brain activities, the calculated exponent of DFA is usually larger than 0.5 but <1.0, implying that the neural processes under investigation have LRTCs (10). Empirical studies in both humans and animals have revealed that the scale-free dynamics of brain activity (i.e., its LRTCs or DFA exponent) could be modulated by neurodegenerative diseases, such as ASD, major depressive disorder, and Alzheimer's disease (11–13). It is assumed that LRTCs are crucial to the efficiency of sustained cognitive operations, including language and social communication, which are impaired in patients with ASD. This theory has been proven by previous studies, which showed that the LRTCs during amplitude modulation of cortical activities were attenuated in patients with ASD (13–15). However, to the best of our knowledge, no study has tested whether the LRTCs of time-varying phase synchrony (TV-PS) of intrinsic brain oscillations could be modulated by ASD.

Here, we investigated whether the LRTCs of TV-PS between electrocortical signals were altered in patients with ASD using DFA on the resting electroencephalogram (EEG) datasets from the healthy brain network (HBN) (16). We hypothesized that the DFA exponents of the TV-PS time series were altered in these patients, especially the phase synchrony between brain regions known to be associated with autism.

## Materials and Methods

### Participants

In the HBN database, 25 ASD patients and 27 typical developing (TD) individuals were selected as participants; note that some of the EEG datasets were used in our previous studies (15, 17). All participants were male, aged between 5 and 18 years (ASD: mean age = 11.5 years, SD = 4 years; TD: mean age = 9.2 years, SD = 2.1 years), right-handed, with an IQ higher than 66. Significant differences between two groups on age and IQ scores were not found (*ps* > 0.05). The diagnosis of ASD was based on the Schedule for Affective Disorders and Schizophrenia-Children's version (18) and the Autism Diagnostic Observation Schedule (ADOS) (1). Note that, the ADOS scores of participants were not available in the current HBN database, thus their ADOS scores were not reported here.

### EEG Recording

During the 5 min resting-state EEG collection, the participants alternately kept their eyes open and closed. Previous study have shown that EEG signal length of ~5 min was enough to produce stable measurements of the DFA exponent (19). A 128-channel Hydro-Cel Geodesic system (EGI Inc., Eugene, Oregon, USA), with an electrode over the vertex of the head as an online reference channel, was used. The ground electrode for the EEG recordings was placed on the forehead. The electrode impedances were kept below 40 kΩ throughout the data recording. The EEG data were recorded using a sampling rate of 500 Hz and a band-pass filtering 0.1–100 Hz. This study was conducted in accordance with the Declaration of Helsinki; ethical approval was obtained from the Chesapeake Institutional Review Board. Written consent was obtained from the participants and their legal guardians (16).

### EEG Data Preprocessing

The EEG data were preprocessed using EEGLAB v13.0.0 (20). The preprocessing consisted of the following steps. Firstly, the electrodes over the neck/face and data portions with large drift were excluded. Secondly, the electrodes with low signal-to-noise ratio (SNR) and large drift longer than 1 min were identified through visual inspection, and were interpolated using a spherical spline method in EEGLAB (function *pop_interp.m*). The large drifts <1 min were deleted after interpolating the above “bad electrodes.” Thirdly, EEG signals were resampled to 250 Hz, band-pass filtered between 0.5 and 80 Hz using Hamming windowed finite impulse response (FIR) filters (function *pop_eegfiltnew.m*). The order of the FIR was chosen to be 1,500, i.e., three cycles of the lower edge of the band considered. A notch filter was used to eliminate 60 Hz line noise. Fourthly, the Infomax independent component analysis (ICA) algorithm (function *pop_runica.m*) was applied to correct physiological artifacts (i.e., eye movements & blinks, electromyography, electrocardiography) and non-physiological artifacts. Lastly, the EEG data were re-referenced to a common average reference.

### Source Localization

The exact low-resolution brain electromagnetic tomography (eLORETA), which can calculate the current density (A/m^2^) of 6,239 cortical gray matter voxels with 5 mm spatial resolution using the MNI152 template, was used to determine the intracerebral electrical source activities from the scalp electrical potentials for each participant (21). Then, the current density time-series of 84 Brodmann areas (Table 1), which were defined as cortical regions of interest (ROIs), were extracted for the following six frequency bands: delta (2–4 Hz), theta (4–8 Hz), alpha (8–13 Hz), beta (14–30 Hz), low-gamma (30–55 Hz), and high-gamma (65–80 Hz), through Hamming windowed FIR filters. The order of the FIR filter was three cycles of the lower edge of each EEG band, that is, the orders for delta, theta, alpha, beta, low-gamma, and high-gamma were 375, 188, 94, 54, 25, and 12, respectively. The above source localization was conducted using the LORETA software (http://www.uzh.ch/keyinst/loreta.htm).

**Table 1:** The 84 ROIs defined in the current study, which were exactly same as Jia and Yu (15).

**ROI**	**Brodmann area**	**Abbrev**.	**Brain regions**
Jan-43	1L/1R	S11	Primary somatosensory cortex 1
Feb-44	2L/2R	S12	Primary somatosensory cortex 2
Mar-45	3L/3R	S13	Primary somatosensory cortex 3
Apr-46	4L/4R	M1	Primary motor cortex
May-47	5L/5R	SPS	Superior parietal sulcus
Jun-48	6L/6R	SMA	Supplementary motor area
Jul-49	7L/7R	PC	precuneus
Aug-50	8L/8R	Pre-SMA	Pre-supplementary motor area
Sep-51	9L/9R	DLPFC	Dorsolateral pre-frontal cortex
Oct-52	10L/10R	FPC	Fronto-parietal cortex
Nov-53	11L/11R	OFC	Orbital frontal cortex
Dec-54	13L/13R	Insula	Insula
13/55	17L/17R	V1	Primary visual cortex
14/56	18L/18R	V2	Secondary visual cortex
15/57	19L/19R	Cuneus	Cuneus
16/58	20L/20R	ITG	Inferior temporal gyrus
17/59	21L/21R	MTG	Medial temporal gyrus
18/60	22L/22R	STG	Superior temporal gyrus
19/61	23L/23R	PCC1	Posterior cingulate cortex 1
20/62	24L/24R	dACC	Dorsal anterior cingulate cortex
21/63	25L/25R	sgACC	Subgeneual anterior cingulate cortex
22/64	27L/27R	PHG1	Parahippocampal gyrus 1
23/65	28L/28R	HIP1	Hippocampal area 1
24/66	29L/29R	RSC1	Retrosplenial cortex 1
25/67	30L/30R	RSC2	Retrosplenial cortex 2
26/68	31L/31R	PCC2	Posterior cingulate cortex 2
27/69	32L/32R	PrACC	Pregeneual anterior cingulate cortex
28/70	33L/33R	rACC	Rostral anterior cingulate cortex
29/71	34L/34R	PHG2	Parahippocampal gyrus 2
30/72	35L/35R	HIP2	Hippocampal area 2
31/73	36L/36R	PHG3	Parahippocampal gyrus 3
32/74	37L/37R	OTC	Occipital-temporal cortex
33/75	38L/38R	TP	Temporal pole
34/76	39L/39R	AG	Angular gyrus
35/77	40L/40R	IPS	Intra-parietal sulcus
36/78	41L/41R	A1	Primary auditory cortex
37/79	42L/42R	A2	Secondary auditory cortex
38/80	43L/43R	PCG	Postcentral gyrus
39/81	44L/44R	OIFG	Opercular part of inferior frontal gyrus
40/82	45L/45R	IFG	Inferior frontal gyrus
41/83	46L/46R	MPFC	Medial prefrontal cortex
42/84	47L/47R	VLPFC	Ventrolateral prefrontal cortex

*R, right hemisphere; L, left hemisphere*.

In the previous data filtering, the alpha band limits were determined to be 8–13 Hz for all participants in both groups. This was based on the fact that the peak alpha frequencies of the occipital electrodes were between 9 and 11 Hz for all participants.

For the gamma band, we studied the low-gamma band (30–55 Hz) and high-gamma band (65–80 Hz). It's well-known that the power of gamma band, especially the high-gamma band, was much lower than other frequency bands and can be easily contaminated by some kinds of artifacts (such as the electromyography), which may lead to inaccurate phase estimation. However, many previous studies have shown that with proper EEG preprocessing and data analysis, it's possible to accurately estimate the power and phase of high-gamma band (22, 23).

### DFA on the Region-to-Region, Time-Varying, Phase Synchrony Time Series

In most of the previous literatures, the term, “*phase synchrony*,”, refers to a fixed phase difference between two signals for a certain duration (24). Full synchronization (i.e., the phase difference between neural oscillators is completely consistent over recording time) or full desynchronisation (i.e., the phase difference between neural oscillators varies randomly over recording time) may indicate a pathological state in humans, whereas the non-fixed yet non-random phase relationships between neurophysiological signals could easily emerge within the normal cortical networks (25). Thus, in the current study, the term, “*phase synchrony*,” is used to describe any pattern of phase relationship between two neuronal oscillations, which could either be fixed or non-fixed values.

Here, the LRTCs of time-varying fluctuations of region-to-region phase synchrony were estimated using the DFA. The procedures for each frequency band and each participant are as follows:

1. The phase time series of neuronal oscillations of each cortical ROI was extracted using the Hilbert transform. Assuming *X*_*H*_(*t*) is the Hilbert transform of the original band-pass filtered source activity *X*(*t*), the time-varying phase ϕ(*t*) can be calculated as: $\phi \left(t\right)=\;{\text{tan}}^{-1}\frac{X_{H}\left(t\right)}{X\left(t\right)}$. The calculated phase time series are limited to a range [–π, π]; discontinuity (e.g., the phase difference between two consecutive time points is larger than or equal to π) can be found in the phase time series. To convert the phase time series into a continuous time series, the phases were unwrapped by adding multiples of ± 2π when discontinuity occurred.
2. Determine the phase differences between the neuronal oscillations in the two cortical ROIs. Note that, since 84 ROIs were defined in the present study, a total of 3,486, that is, $C_{84}^{2},$ phase difference time series were calculated for each frequency band and participant.
3. Compute the rate of change of the phase-difference time series. Because the unwrapped phase time series will continue to evolve as time increases, their phase difference time series are unbounded. However, a DFA is typically applicable to bounded processes. Consistent with previous studies, phase synchrony was quantified as the rate of change of the phase difference time series, that is, its first-time derivative (25).
4. The signal profile of the rate of change of the phase difference time series (i.e., its cumulative sum) was computed for each pair of neuronal oscillations.
5. Each signal profile was divided into dozens of windows with length τ and 50% overlap. The set of window length τ was between 1 and 15 s (for alpha, beta, low-gamma, and high-gamma bands) or between 2 and 15 s (for delta and theta bands) equidistantly on a logarithmic scale. The linear trend was detrended by subtracting a trendline determined by a least-squares fit. The standard deviation of each detrended window was then calculated. Finally, the fluctuation function for window length τ was computed, that is, the mean value of standard deviations across all detrended segmented signal profiles with length τ.

The scatter plot between logarithmic-transformed window lengths and logarithmic-transformed fluctuation functions was defined as *a DFA fluctuation plot*. The slope of the least-squares line for this scatter plot, which is referred to as the DFA exponent in the literature, provides an estimation of the LRTCs or scale-free property of a given region-to-region TV-PS time series. The linear scaling in log space was confirmed by the high value of the coefficient of determination (i.e., *R*^2^ > 0.9 for most of the TV-PS time series) and stringent validation tests, as shown below. The above DFA exponent calculation was conducted using the Neurophysiological Biomarker Toolbox (https://github.com/NBT-Analytics/NBTpublic).

### Validating the Presence of Scale-Invariance

The DFA exponent estimation was based on the assumption of linearity in the DFA fluctuation plots. This assumption was verified by a maximum likelihood (ML)-based model selection technique (ML-DFA). In this technique, we fitted the DFA fluctuation plots with 13 alternative models (i.e., linear, quadratic, cubic, quartic, quantic, square root, cube root, fourth root, exponential, logarithmic, and spline with 2–4 linear sections). The Akaike information criterion (AIC) that traded off the goodness-of-fit against the number of parameters was then estimated for each model. If the linear model had the lowest AIC value compared to the other models, the linearity in the DFA fluctuation plots and the scale-invariance of the original TV-PS time series were validated (26). The MATLAB function *ML_DFA.m* which was used to validate the presence of scale-invariance can be downloaded from http://users.sussex.ac.uk/~lb203/Software/assets/MLDFA-30092013.zip.

To evaluate the presence of scale-invariance in the TV-PS time series, the percentage of participants accepting the existing linear scaling was computed for each ROI pair, frequency band, and group.

### Statistical Tests

To examine whether the LRTCs of the TV-PS time series were significantly altered in patients with ASD, the network-based statistic (NBS) was performed for the DFA exponent at each frequency band. Using an approach similar to the cluster-based permutation test used in the activation tests, the NBS is a non-parametric, statistical method to avoid the multiple comparison problem encountered when conducting mass univariate statistical testing in functional networks (27). This method consists of the following steps (27): First, F-statistics for all the 3,486 ($C_{84}^{2}$) edges on the graph based on the differences in DFA exponent values between groups, with the age of participants included as a covariate, were computed. In NBS, the statistical model is specified in terms of the general linear model (GLM). Here, the covariate was included as nuisance regressor in the design matrix of GLM. Second, a primary threshold (*p* < 0.005, uncorrected) was used to identify all edges displaying potential differences in the DFA exponent value. From these identified edges, the so-called, “connected graph components,” which were defined as a set of supra-threshold edges for which a path could be found between any two cortical ROIs, were identified. Third, the size of each connected graph component was defined as the total number of edges it comprises (i.e., “component extent”). In NBS, the component size could also be measured by the sum of test statistic values across all connections comprising the component. This is referred to as the component intensity. In the current study, these two approaches revealed exactly the same results. Fourth, the null distribution of the size of the connected graph component was empirically derived using a permutation approach with 5,000 permutations. For each permutation, all participants were randomly allocated into two groups; the above three steps were then conducted. The component with a maximum cluster size was noted for each permutation, which yielded an empirical null distribution for the largest component size. Lastly, the one-sided, family wise error rate (FWER)-corrected *p*-value for an originally identified component was estimated as the proportion of permutations for which the largest component was of equal size or greater. The final results controlled the FWER (weak sense) at the cluster level (*p* < 0.05).

The statistical tests were conducted using the MATLAB toolbox NBS v1.2 (https://www.nitrc.org/projects/nbs/).

## Results

### The Presence of Scale-Invariance

**Figure 1:**
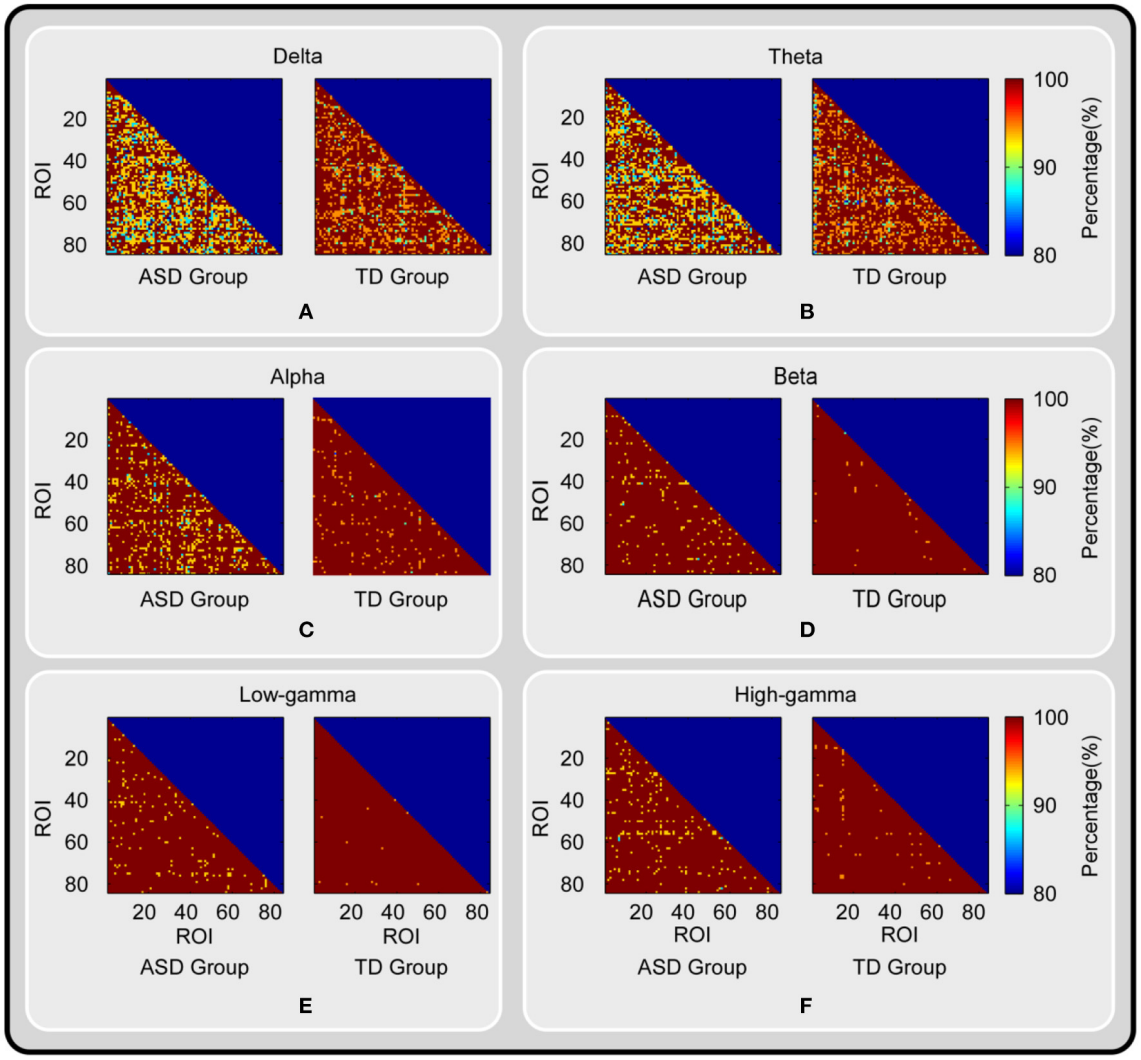
The results of ML-DFA conducted on the TV-PS time series between brain oscillations. The percentage of participants for which the presence of scale-invariance was accepted for each ROI pair, each group and each frequency band is shown. The results for delta, theta, alpha, beta, low-gamma and high-gamma band are color coded and presented in panel **(A-F)** respectively.

The results of the ML-DFA conducted on the region-to-region TV-PS time series are shown in Figure 1. We found that for most ROI pairs, the percentage of participants for which the presence of scale-invariance was accepted was higher than 90%. Moreover, for most ROI pairs in the beta, low-gamma, and high-gamma bands, this percentage reached 100% in the TD group. Additionally, this percentage was much higher in higher frequency bands (i.e., alpha, beta, low-gamma, and high-gamma bands) than in the lower frequency bands (i.e., delta and theta bands), and was much higher in the TD group than in the ASD group.

### The Group Effect on DFA Exponents

**Figure 2:**
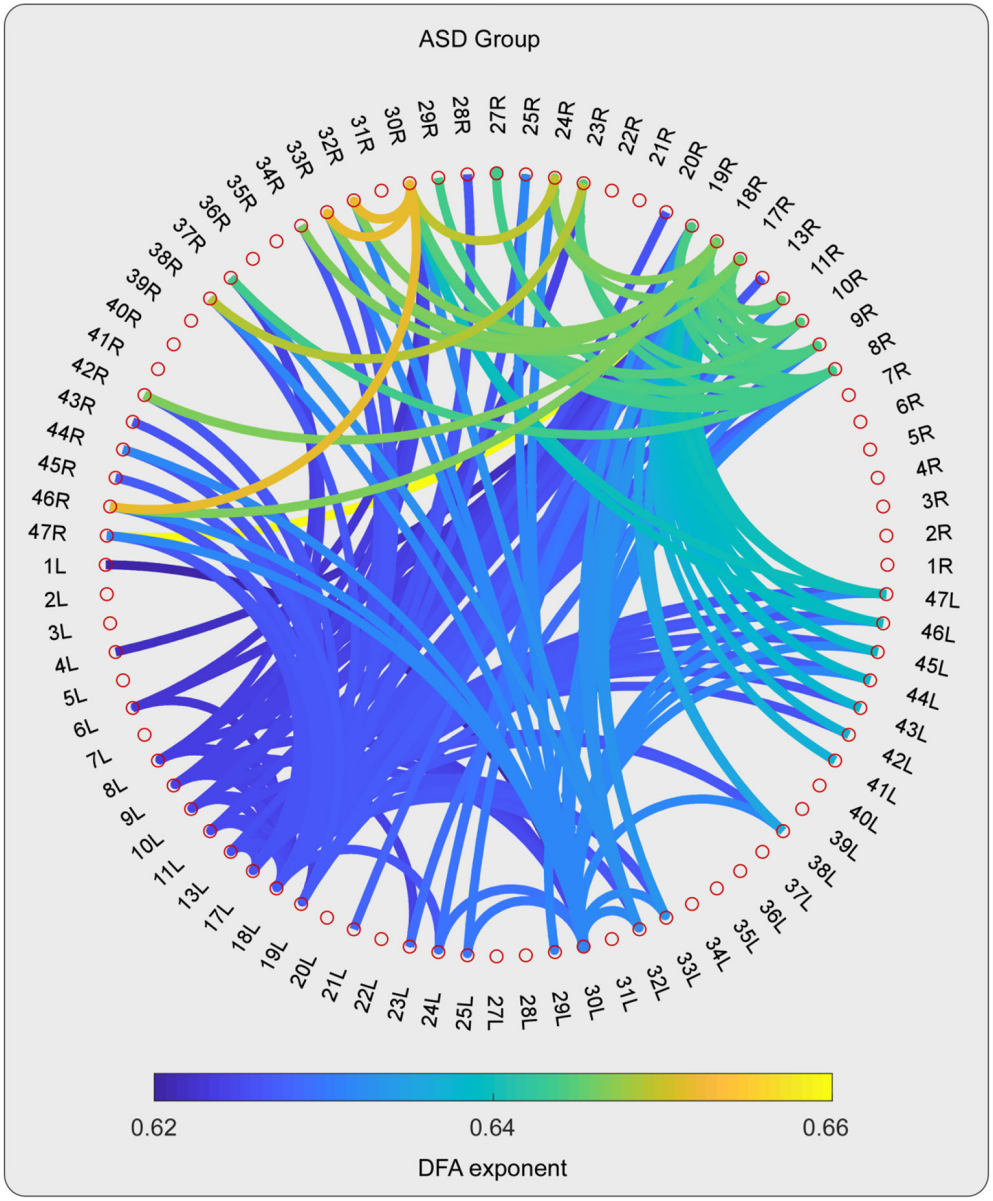
The DFA exponents of TV-PS time series in high-gamma oscillations for ASD group. The magnitude of DFA exponent is color coded. Note that, only the pairs with 10% largest DFA exponent are shown.

**Figure 3:**
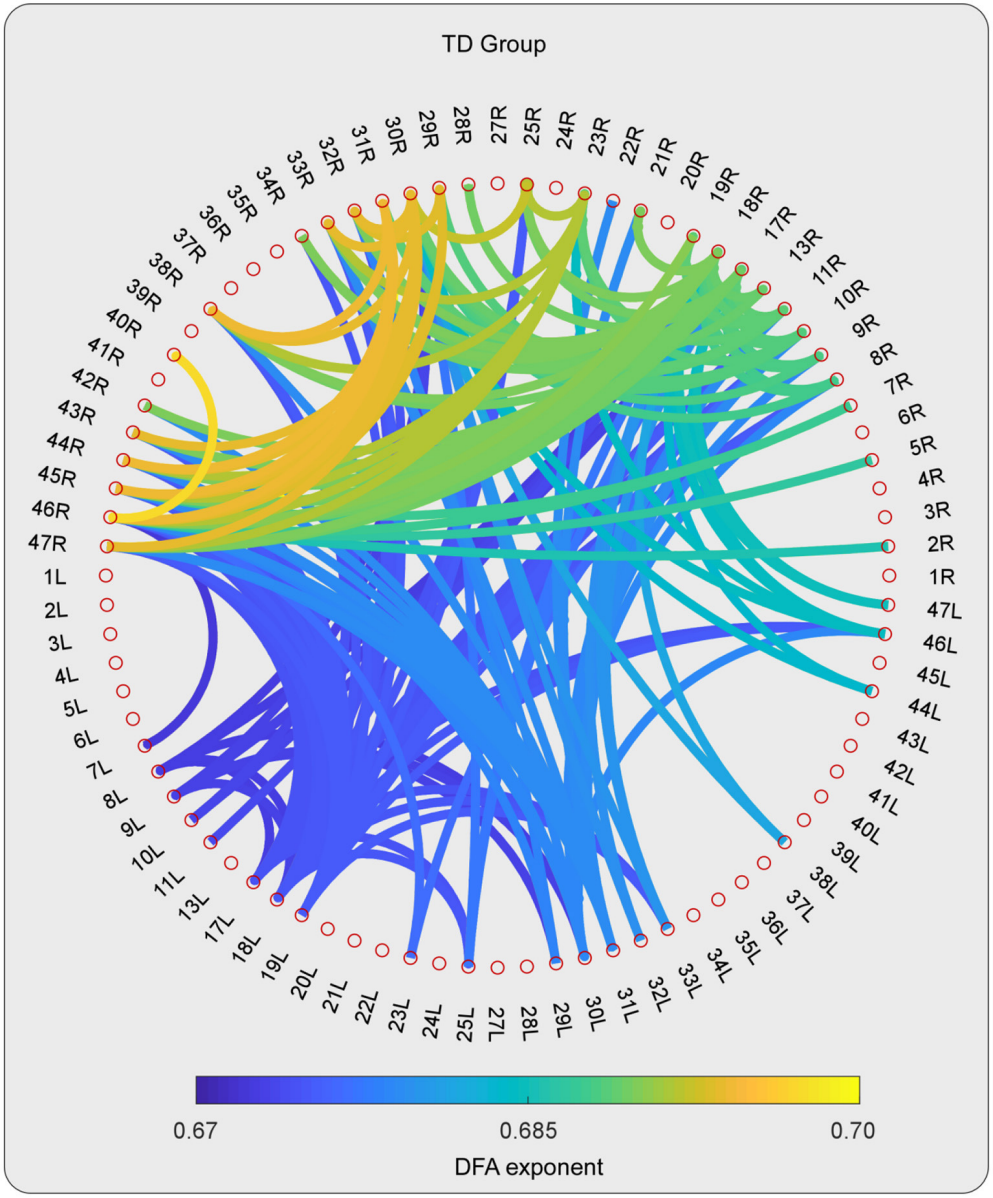
The DFA exponents of TV-PS time series in high-gamma oscillations for TD group. The magnitude of DFA exponent is color coded. Note that, only the pairs with 10% largest DFA exponent are shown.

**Figure 4:**
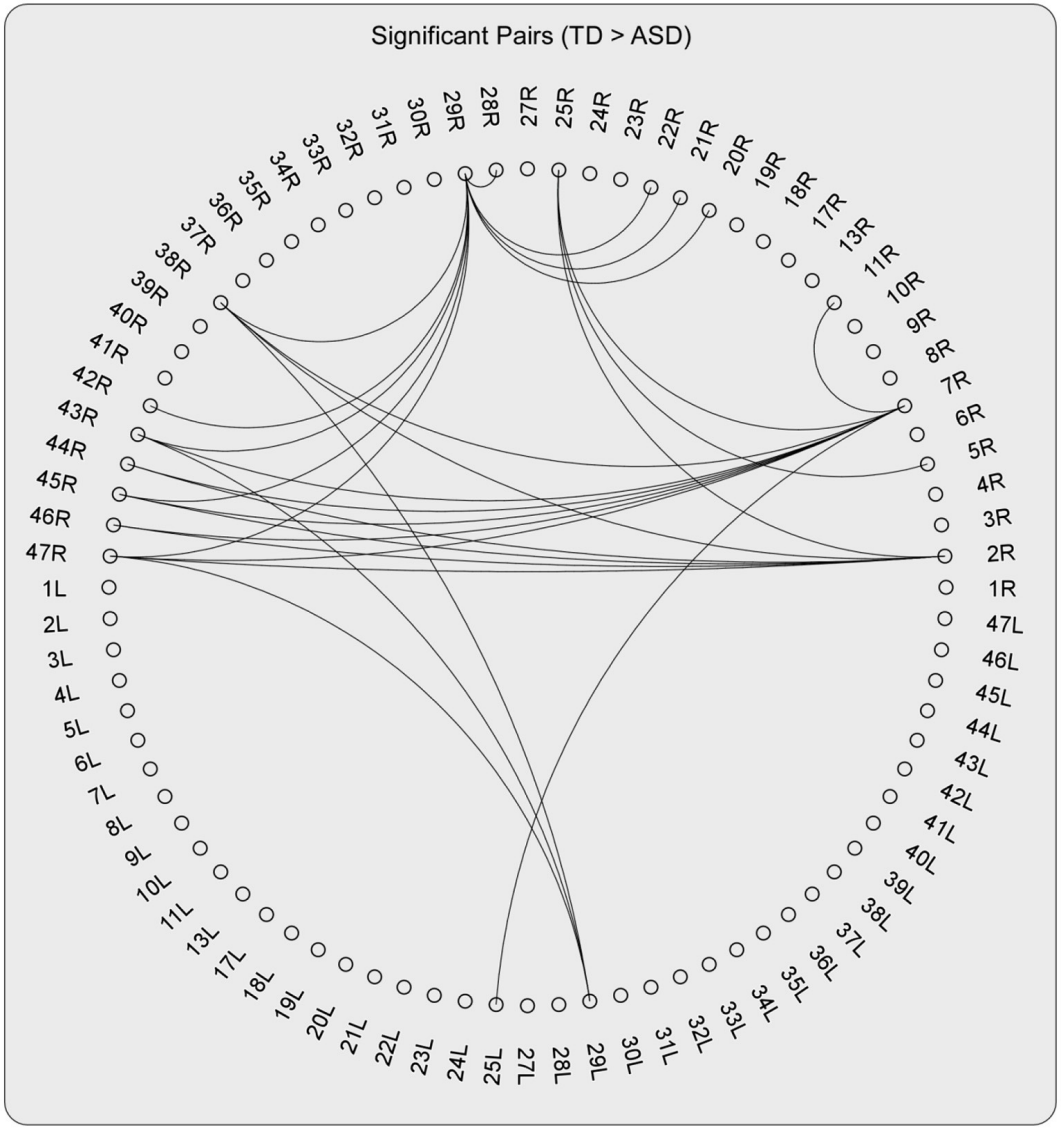
The pairs with significant group effect (TD > ASD) when testing the group differences of the DFA exponents of TV-PS time series in high-gamma oscillations.

For the high-gamma band, the statistical tests on the DFA exponents showed that those in 28 ROI pairs in the TD group were significantly larger than those in the ASD group (Figures 2–**4**). No significant results were found for the other frequency bands. The NBS also did not detect any significant results when the alternative hypothesis was ASD > TD.

As can be detected in Figures 4, **6**, The ROIs involved in aberrant TV-PS time series were mainly located in the right hemisphere, including 2R (primary somatosensory cortex1), 5R (superior parietal sulcus), 7R (precuneus), 11R (orbital frontal cortex), 20R (inferior temporal gyrus), 21R (medial temporal gyrus), 22R (superior temporal gyrus), 25R (subgeneual anterior cingulate cortex), 28R (hippocampal area), 29R (retrosplenial cortex1), 38R (temporal pole), 42R (secondary auditory cortex), 43R (postcentral gyrus), 44R (opercular part of inferior frontal gyrus), 45R (inferior frontal gyrus), 46R (medial prefrontal cortex), and 47R (ventrolateral prefrontal cortex). Only two cortical ROIs involved in aberrant TV-PS time series were found in the left hemisphere; they were 25 L (subgenetic anterior cingulate cortex) and 29 L (retrosplenial cortex1).

**Figure 5:**
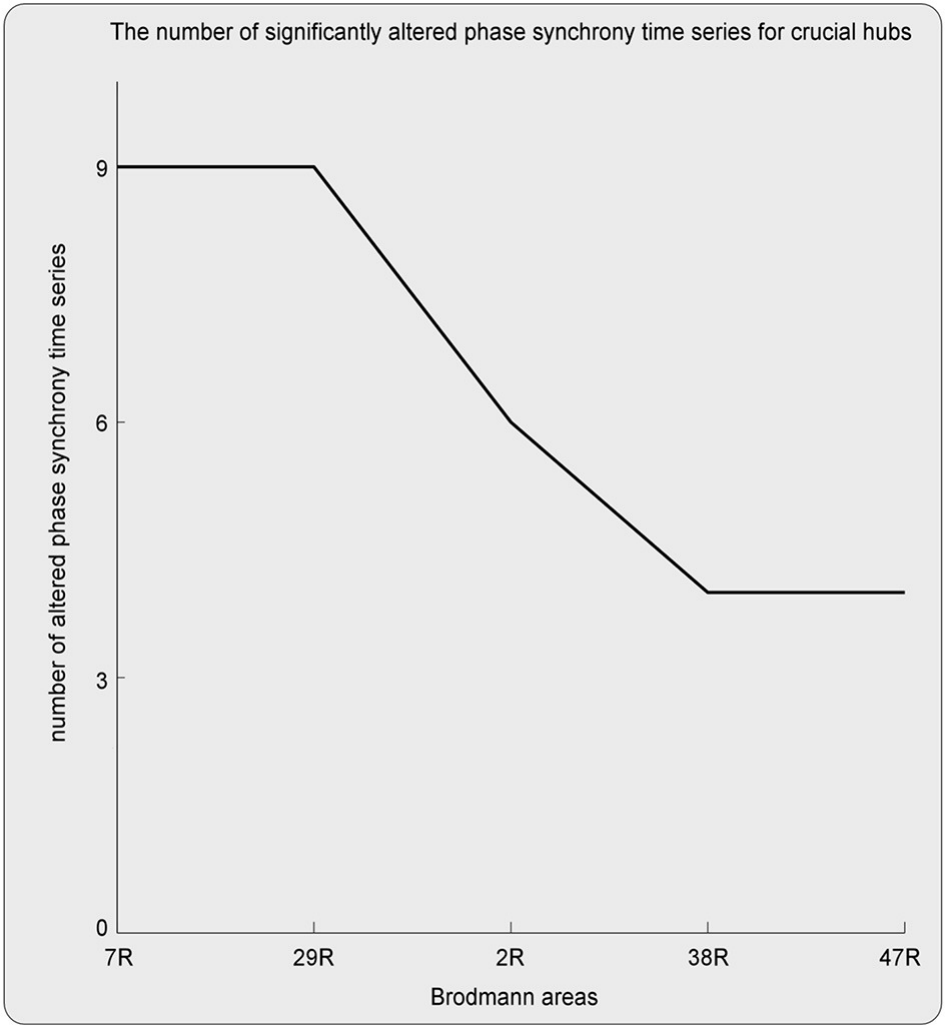
The crucial hubs (i.e., the ROIs with more than four aberrant TV-PS time series) and the number of significantly altered TV-PS time series for these crucial hubs.

**Figure 6:**
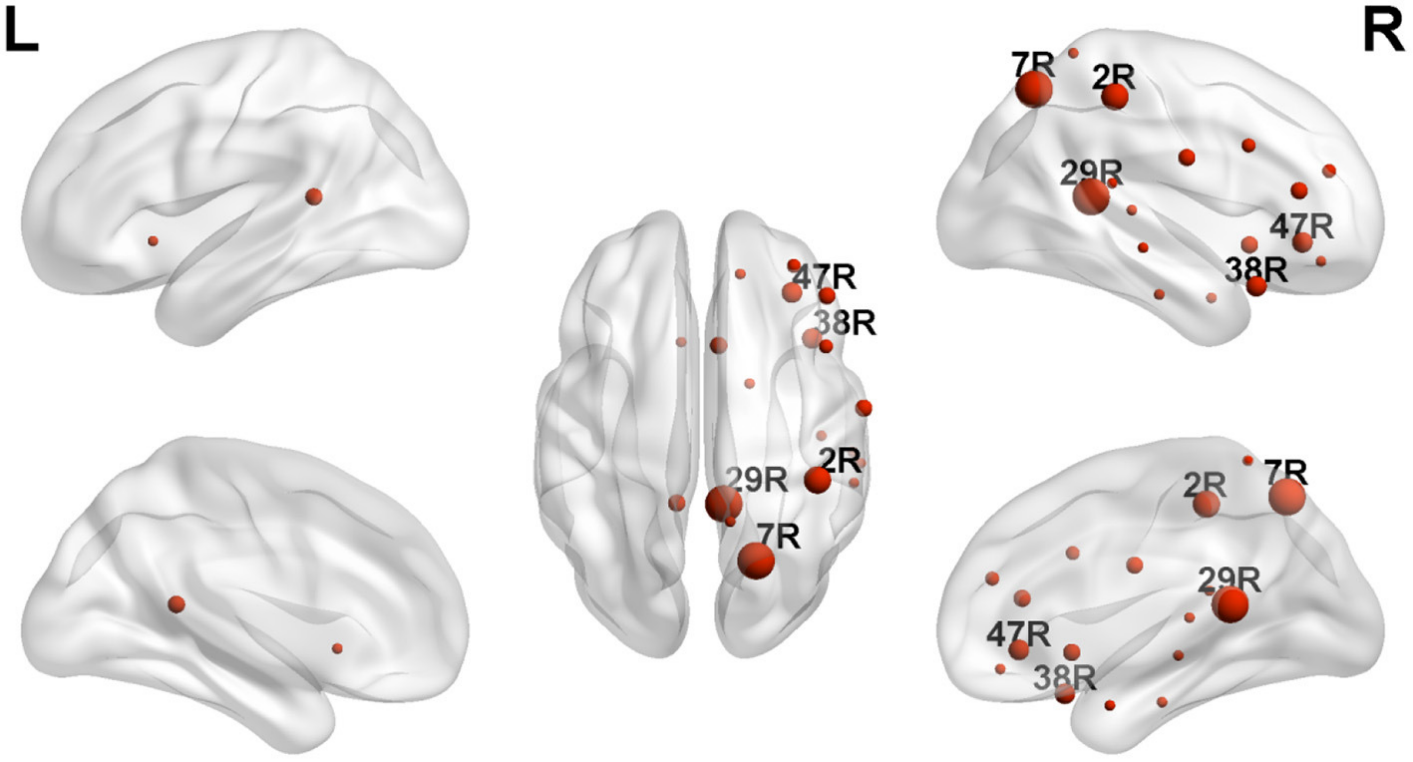
The locations of ROIs with significant group effect (TD > ASD) revealed by NBS. The size of each ROI corresponds to its number of significantly altered TV-PS time series. Only the labels of crucial hubs were shown.

Here, ROIs with more than four aberrant phase synchrony time series were identified as “crucial hubs” (Figures 5, 6); they were 7R (precuneus), 29R (retrosplenial cortex1), 2R (primary somatosensory cortex1), 38R (temporal pole), and 47R (ventrolateral prefrontal cortex). These ROIs accounted for 26 aberrant phase synchrony time series. Only two aberrant phase synchrony time series (i.e., 5R−25R and 43R−29L) did not involve these crucial hubs.

Because the magnitude (i.e., power spectra) of the neuronal oscillations of each frequency band could influence the successful estimation of the phase time series and of the DFA exponents, we tested whether significant group differences in the power spectra of each frequency band could be detected. The results showed that significant group differences could not be detected in all six bands and ROIs.

## Discussion

Here, we investigated whether the LRTCs of the region-to-region TV-PS time series were significantly altered in patients with ASD. First, the results of the ML-DFA confirmed the presence of scale invariance in the TV-PS time series. Second, the LRTCs of TV-PS within the high-gamma band were found to be reduced in these patients.

### The Scale-Free Dynamics and LRTCs in Brain Oscillations

Exploring the datasets in behavioral science and neuroscience, previous researchers found that scale invariance/LRTCs are omnipresent in nature, which supports the theory that cortical activities may sit in a dynamic state close to the criticality (28). The advantages of criticality demonstrated through computational simulations and experimental studies include the following aspects: (1) the maximum dynamic range of information processing/communication and its efficiency within systems, and (2) a readiness to respond to environmental changes (10, 25). The presence of scale-invariance, which was validated by ML-DFA, was observed in most of the TV-PS time series across all the region-to-region pairs, the six frequency bands investigated, and the participants in both groups. Moreover, in Figure 1, we found that the percentage of participants in the TD group for which the presence of scale-invariance was accepted was larger than that of the ASD group for all six frequency bands and nearly all the ROI pairs. Thus, the results detected may suggest that although scale-invariant dynamics were inherent in cortical-cortical TV-PS time series of autistic brains, this property may be vulnerable in these patients.

Scale-free dynamics and LRTCs in neuronal oscillations are associated with the efficiency of certain operations (e.g., learning, memory processes, and information transfer) within the brain system (25). Several studies have demonstrated the presence of LRTCs and scale invariance for the TV-PS between pairs of neural oscillations and found that this property of phase synchrony could be influenced by some experimental operations, such as finger movement (25, 29). Previous studies have indicated that the magnitude of LRTCs is positively correlated with the ability of the human brain to maintain transiently stable oscillations in support of active neuronal representations during sustained cognitive operations (e.g., language, social cognition, and communication), which are impaired in patients with ASD (30). Moreover, it has been widely revealed that aberrant functional connectivity is inherent in an autistic brain (31). Thus, we investigated whether the LRTCs of phase synchrony were disrupted in autistic brains using source-level electrocortical oscillations, which could provide rich temporal, spectral, and spatial information about brain activities.

### The Attenuated LRTCs of Time-Varying Phase Synchrony in Autistic Brain

The statistical tests on the DFA exponent showed that those in 28 ROI pairs in the ASD group were significantly smaller than those in the TD group for the high-gamma band. Cortical regions involved in the aberrant phase synchrony time series were mainly located in the right hemisphere.

Here, the ROIs that explained 26 out of 28 aberrant connections and with more than four aberrant phase synchrony time series were defined as “crucial hubs.” These brain areas were the precuneus, retrosplenial cortex (RSC), primary somatosensory cortex (S1), temporal pole (TP), and ventrolateral prefrontal cortex (VLPFC). The precuneus, located in the posteromedial portion of the parietal lobe, is involved in visuo-spatial imagery, episodic memory retrieval, and self-processing operations (32). The RSC is closely associated with a wide range of brain functions, such as episodic memory, navigation, imagination, and planning for the future (33). The TP plays a crucial role in both social and emotional processes, such as face recognition and theory of mind (34–36). Specifically, the right TP is believed to be the storehouse or site of recollection of personal, episodic memories, and is involved in high-level sensory representations with emotional responses and social memory (34). These three regions are the main hubs of the default mode network (DMN) and are suggested to be involved in the pathophysiology of autism (2, 13). For S1, previous studies have shown that this region often exhibits atypical responses to touch in patients with ASD (37). Moreover, since S1 is a crucial region in the mirror neuron system (MNS), it may also closely modulate social cognition and interaction in patients with ASD. The VLPFC, especially the right VLPFC, plays an important role in emotional regulation in the social context (38). It is also an important cortical region of the MNS that underlies the dysfunction of social cognition in autism (39). Almost all the aberrant DFA exponents (i.e., 26 of 28) could be explained by the connections between these five hubs and other brain regions. It should be noted that these results may also be caused by the fact that these five brain regions manifested as certain specific patterns (e.g., periodic activity) in the autistic brain, which requires further investigation.

The presence of scale-free dynamics has been found in the moment-to-moment fluctuations of phase synchrony; the healthy resting brain state is accompanied by weak and variable neural synchrony which shows slow fluctuations over time (29). The LRTCs of the TV-PS time series may reflect a state of readiness that facilitates rapid task-dependent shifts toward and away from full synchronization or full desynchronisation (25). Thus, the attenuated LRTCs of phase synchrony between certain regions in patients with ASD may indicate that the autistic brain, especially those regions involved in autism, deviated from an optimum state of readiness to external or internal events.

In a study conducted by our team (15), the DFA exponents of the instantaneous amplitude of the beta and low-gamma oscillations of DMN, MNS, and salience network (SN) were significantly attenuated in patients with ASD, as compared with the TD participants. First, the regions with altered DFA exponents (i.e., LRTCs) were highly overlapped between DFA conducted on the instantaneous amplitude time series and DFA conducted on the phase synchrony time series. Second, compared with DFA conducted on the instantaneous amplitude time series, the EEG band with significant results turned into a high-gamma band when TV-PS was investigated. (40) found that temporal changes in phase synchrony exhibit less temporally structured and more complex correlations, indicating fast and flexible coding (40). This could be the reason why the frequencies showing aberrant LRTCs occur at much faster rates (i.e., high-gamma) when the phase synchrony time series were investigated.

Here, nearly all the aberrant phase synchrony time series were caused by cortical regions over the right hemisphere. Atypical laterality using neuroimaging techniques has been widely studied in the ASD group (41). For the EEG gamma band, previous studies showed that the resting gamma power was reduced in patients with ASD and was specific to the right lateral hemisphere (42). Note that none of the previous studies have investigated the LRTCs of phase synchrony time series in patients with psychiatric disorders. Thus, this is the first study to show that abnormal laterality of LRTCs could be seen in the phase synchrony between cortical regions in patients with ASD.

It should be mentioned that the largest window size used in the current study was 15 s. If the largest window size was increased (e.g., 20 s), the percentage of fluctuation plots being accepted as linear and the value of DFA exponents may be decreased. However, in an exploratory analysis, we found that if the largest window size was set to 20 s, the results of the group comparison were similar to those reported here.

### Comparison With Other Studies Focused on the Time-Varying Features in Autistic Brain

In these years, more and more studies investigated various aspects of time-varying neural features in ASD. For example, Sase and Kitajo investigated the metastability in the brain oscillations through the phase-phase coupling (PPC) and phase-amplitude coupling (PAC) of resting-state EEG signals in typically developing individuals with different levels of autistic-like traits (43). They found that the metastable dynamics of synchronization and amplitude modulation is correlated with levels of autistic traits. Moreover, fewer transitions between states occurred in individuals with relatively longer attention span. In the other study, using resting-state fMRI signals, Watanabe and Rees characterized the cortical dynamics in autism through an energy-landscape analysis (44). They found that patients with ASD show fewer neural transitions between two major brain states, and this abnormal transition can predict the severity of autism. Here, we investigated the scale-invariant characters of resting-state EEG signals in autism, and found that the LRTCs of region-to-region, time-varying, phase synchrony time series within the high-gamma band were reduced in the autistic brain. Due to the complexity of time-varying neural features in human brain, more studies are needed in order to obtain a comprehensive understanding of neural dynamics in autism.

### The Limitations of the Current Study

Several limitations of the current study should be mentioned. Firstly, the ADOS scores of participants were not available in the current HBN database, thus their ADOS scores were not reported and analyzed here. Without these scores, we could not assess the associations between the severity of symptoms and DFA exponents, which limited the reliability and interpretation of the findings as well as the guidance for clinical treatment and intervention. Secondly, during the validation of the presence of scale-invariance of TV-PS time series, we only showed some descriptive measures (e.g., the percentage of participants for which the presence of scale-invariance) in Figure 1. We did not conduct any statistical tests between groups, which may limit the interpretation of the findings between groups.

## Conclusion

In the present study, we found that the LRTCs of region-to-region, time-varying, phase synchrony time series within the high-gamma band were reduced in the autistic brain. Furthermore, the cortical regions involved in aberrant LRTCs are mainly located in well-recognized brain networks associated with autism. These results indicate that reduced LRTCs of time-varying phase synchrony within high-gamma oscillations may play an important role in the dysfunction of social, communication, and emotional abilities commonly observed in patients with ASD.

## Data Availability Statement

Publicly available datasets were analyzed in this study. This data can be found here: http://fcon_1000.projects.nitrc.org/indi/cmi_healthy_brain_network.

## Ethics Statement

The studies involving human participants were reviewed and approved by Chesapeake Institutional Review Board. Written informed consent to participate in this study was provided by the participants' legal guardian/next of kin.

## Author Contributions

The work presented here was carried out in collaboration between all authors. HJ and FG initiated and analyzed the data and wrote the manuscript. DY wrote and revised the manuscript. All authors have read and approved the final published manuscript.

## Funding

This work was supported by the Philosophy and Social Sciences Planning Project of Henan Province under Grant No. 2020BJY010 and Henan University Philosophy and Social Science Innovation Team under Grant 2019CXTD009.

## Conflict of Interest

The authors declare that the research was conducted in the absence of any commercial or financial relationships that could be construed as a potential conflict of interest. The reviewer LS declared a shared affiliation, with no collaboration, with the author FG at the time of the review.

## Publisher's Note

All claims expressed in this article are solely those of the authors and do not necessarily represent those of their affiliated organizations, or those of the publisher, the editors and the reviewers. Any product that may be evaluated in this article, or claim that may be made by its manufacturer, is not guaranteed or endorsed by the publisher.

## References

[1] Lord C Rutter M DiLavore PC Risi S Gotham K Bishop S. Autism Diagnostic Observation Schedule (ADOS-2). 2nd ed. Torrence, CA: Western Psychological Corporation (2012).

[2] Padmanabhan A Lynch CJ Schaer M Menon V. The Default Mode Network in Autism. Biol Psychiatry Cogn Neurosci Neuroimaging. (2017) 2:476–86. 10.1016/j.bpsc.2017.04.004 29034353 PMC5635856

[3] Amaral DG Schumann CM Nordahl CW. Neuroanatomy of autism. Trends Neurosci. (2008) 31:137–45. 10.1016/j.tins.2007.12.005 18258309

[4] Belmonte MK Allen G Beckelmitchener A Boulanger LM Carper RA Webb SJ. Autism and abnormal development of brain connectivity. J Neurosci. (2004) 24:9228–31. 10.1523/JNEUROSCI.3340-04.2004 15496656 PMC6730085

[5] Hutchison RM Womelsdorf T Allen EA Bandettini PA Calhoun VD Corbetta M. Dynamic functional connectivity: Promise, issues, and interpretations. Neuroimage. (2013) 80:360–78. 10.1016/j.neuroimage.2013.05.079 23707587 PMC3807588

[6] Falahpour M Thompson WK Abbott AE Jahedi A Mulvey ME Datko M. Underconnected, but not broken? Dynamic functional connectivity mri shows underconnectivity in autism is linked to increased intra-individual variability across time. Brain Connect. (2016) 6:403–14. 10.1089/brain.2015.0389 26973154 PMC4913487

[7] Irrmischer M Houtman SJ Mansvelder HD Tremmel M Ott U Linkenkaer-Hansen K. Controlling the temporal structure of brain oscillations by focused attention meditation. Hum Brain Mapp. (2018) 39:1825–38. 10.1002/hbm.23971 29331064 PMC6585826

[8] Krzeminski D Kaminski M Marchewka A Bola M. Breakdown of long-range temporal correlations in brain oscillations during general anesthesia. Neuroimage. (2017) 159:146–58. 10.1016/j.neuroimage.2017.07.047 28750775

[9] Hardstone R Poil SS Schiavone G Jansen R Nikulin VV Mansvelder HD. Detrended fuctuation analysis: a scale-free view on neuronal oscillations. Front Physiol. (2012) 3:450. 10.3389/fphys.2012.00450 23226132 PMC3510427

[10] Linkenkaer-Hansen K Nikouline VV Palva JM Ilmoniemi RJ. Long-range temporal correlations and scaling behavior in human brain oscillations. Journal of Neuroscience. (2001) 21:1370–7. 10.1523/JNEUROSCI.21-04-01370.2001 11160408 PMC6762238

[11] Linkenkaer-Hansen K Monto S Rytsälä H Suominen K Isometsä E Kähkönen S. Breakdown of long-range temporal correlations in theta oscillations in patients with major depressive disorder. Journal of Neuroscience. (2005) 25:10131–7. 10.1523/JNEUROSCI.3244-05.2005 16267220 PMC6725784

[12] Montez T Poil SS Jones BF Manshanden I Verbunt JP van Dijk BW. Altered temporal correlations in parietal alpha and prefrontal theta oscillations in early-stage Alzheimer disease. Proc Natl Acad Sci USA. (2009) 106:1614–9. 10.1073/pnas.0811699106 19164579 PMC2635782

[13] Lai MC Lombardo MV Chakrabarti B Sadek SA Pasco G Wheelwright SJ. A shift to randomness of brain oscillations in people with autism. Biol Psychiatry. (2010) 68:1092–9. 10.1016/j.biopsych.2010.06.027 20728872

[14] Jia H Li Y Yu D. Attenuation of long-range temporal correlations of neuronal oscillations in young children with autism spectrum disorder. Neuroimage: Clinical. (2018) 20:424–32. 10.1016/j.nicl.2018.08.012 30128281 PMC6095951

[15] Jia H Yu D. Attenuated long-range temporal correlations of electrocortical oscillations in patients with autism spectrum disorder. Developmental Cognitive Neuroence. (2019) 39:100687. 10.1016/j.dcn.2019.100687 31377569 PMC6969363

[16] Alexander LM Escalera J Ai L Andreotti C Febre K Mangone A. An open resource for transdiagnostic research in pediatric mental health and learning disorders. Scientific Data. (2017) 4:170181. 10.1038/sdata.2017.181 29257126 PMC5735921

[17] Jia H Yu D. Aberrant intrinsic brain activity in patients with autism spectrum disorder: insights from EEG microstates. Brain Topogr. (2019) 32:295–303. 10.1007/s10548-018-0685-0 30382452

[18] Kaufman J Birmaher B Brent D Rao U Flynn C Moreci P. Schedule for affective disorders and schizophrenia for school-age children-present and lifetime version (K-SADS-PL): initial reliability and validity data. J Am Acad Child Adolesc Psychiatry. (1997) 36:980–8. 10.1097/00004583-199707000-00021 9204677

[19] Smith RJ Ombao HC Shrey DW Lopour BA. Inference on long-range temporal correlations in human EEG data. IEEE J Biomed Health Inform. (2019) 24:1070–9. 10.1109/JBHI.2019.2936326 31478876

[20] Delorme A Makeig S EEGLAB. an open source toolbox for analysis of single-trial EEG dynamics including independent component analysis. J Neurosci Methods. (2004) 134:9–21. 10.1016/j.jneumeth.2003.10.009 15102499

[21] Pascual-Marqui RD Lehmann D Koukkou M Kochi K Anderer P Saletu B. Assessing interactions in the brain with exact low-resolution electromagnetic tomography. Philos Trans A Math Phys Eng Sci. (2011) 369:3768–84. 10.1098/rsta.2011.0081 21893527

[22] Werkle-Bergner M Shing YL Müller V Li S-C Lindenberger U. EEG gamma-band synchronization in visual coding from childhood to old age: evidence from evoked power and inter-trial phase locking. Clin Neurophysio. (2009) 120:1291–302. 10.1016/j.clinph.2009.04.012 19482545

[23] Yang K Tong L Shu J Zhuang N Yan B Zeng Y. High gamma band eeg closely related to emotion: evidence from functional network. Front Hum Neurosci. (2020) 14:89. 10.3389/fnhum.2020.00089 32265674 PMC7107011

[24] Yu D. Additional brain functional network in adults with attention-deficit/hyperactivity disorder: a phase synchrony analysis. PLoS ONE. (2013) 8:e54516. 10.1371/journal.pone.0054516 23382908 PMC3561351

[25] Botcharova M Farmer SF Berthouze L. Markers of criticality in phase synchronization. Front Syst Neurosci. (2014) 8:176. 10.3389/fnsys.2014.00176 25309353 PMC4173811

[26] Botcharova M Farmer SF Berthouze L. A maximum likelihood based technique for validating detrended fluctuation analysis (ML-DFA). arXiv [Preprint]. (2013). arXiv:1306.5075

[27] Zalesky A Fornito A Bullmore ET. Network-based statistic: identifying differences in brain networks. Neuroimage. (2010) 53:1197–207. 10.1016/j.neuroimage.2010.06.041 20600983

[28] Palva JM Zhigalov A Hirvonen J Korhonen O Linkenkaer-Hansen K Palva S. Neuronal long-range temporal correlations and avalanche dynamics are correlated with behavioral scaling laws. Proc Natl Acad Sci USA. (2013) 110:3585–90. 10.1073/pnas.1216855110 23401536 PMC3587255

[29] Botcharova M Berthouze L Brookes MJ Barnes GR Farmer SF. Resting state MEG oscillations show long-range temporal correlations of phase synchrony that break down during finger movement. Front Physiol. (2015) 6:183. 10.3389/fphys.2015.00183 26136690 PMC4469817

[30] Smit DJA De Geus EJC Van De Nieuwenhuijzen ME Van Beijsterveldt CEM Van Baal GCM Mansvelder HD. Scale-free modulation of resting-state neuronal oscillations reflects prolonged brain maturation in humans. J Neurosci. (2011) 31:13128–36. 10.1523/JNEUROSCI.1678-11.2011 21917796 PMC6623247

[31] Abrams DA Lynch CJ Cheng KM Phillips J Supekar K Ryali S. Underconnectivity between voice-selective cortex and reward circuitry in children with autism. Proc Natl Acad Sci USA. (2013) 110:12060–5. 10.1073/pnas.1302982110 23776244 PMC3718181

[32] Cavanna AE Trimble MR. The precuneus: a review of its functional anatomy and behavioural correlates. Brain. (2006) 129:564–83. 10.1093/brain/awl004 16399806

[33] Vann SD Aggleton JP Maguire EA. What does the retrosplenial cortex do? Nat Rev Neurosci. (2009) 10:792–802. 10.1038/nrn2733 19812579

[34] Olson IR Plotzker A Ezzyat Y. The Enigmatic temporal pole: a review of findings on social and emotional processing. Brain. (2007) 130:1718–31. 10.1093/brain/awm052 17392317

[35] Tsukiura T Mochizuki-Kawai H Fujii T. Dissociable roles of the bilateral anterior temporal lobe in face-name associations: an event-related fMRI study. Neuroimage. (2006) 30:617–26. 10.1016/j.neuroimage.2005.09.043 16275140

[36] Grèzes J Frith C Passingham RE. Brain mechanisms for inferring deceit in the actions of others. J Neurosci. (2004) 24:5500–5. 10.1523/JNEUROSCI.0219-04.2004 15201322 PMC6729335

[37] Kaiser MD Yang DYJ Voos AC Bennett RH Gordon I Pretzsch C. Brain Mechanisms for Processing Affective (and Nonaffective) Touch Are Atypical in Autism. Cerebral Cortex. (2016) 26:2705–14. 10.1093/cercor/bhv125 26048952 PMC4869810

[38] He Z Lin Y Xia L Liu Z Zhang D Elliott R. Critical role of the right VLPFC in emotional regulation of social exclusion: a tDCS study. Soc Cogn Affect Neurosci. (2018) 13:357–66. 10.1093/scan/nsy026 29618116 PMC5928413

[39] Pinkham AE Hopfinger JB Pelphrey KA Piven J Penn DL. Neural Bases for Impaired Social Cognition in Schizophrenia and Autism Spectrum Disorders. Schizophr Res. (2008) 99:164–75. 10.1016/j.schres.2007.10.024 18053686 PMC2740744

[40] Daffertshofer A Ton R Kringelbach ML Woolrich M Deco G. Distinct criticality of phase and amplitude dynamics in the resting brain. Neuroimage. (2018) 180:442–7. 10.1016/j.neuroimage.2018.03.002 29526743

[41] Stroganova TA Nygren G Tsetlin MM Posikera IN Gillberg C Elam M. Abnormal EEG lateralization in boys with autism. Clin Neurophysiol. (2007) 118:1842–54. 10.1016/j.clinph.2007.05.005 17581774

[42] Maxwell CR Villalobos ME Schultz RT Herpertz-Dahlmann B Konrad K Kohls G. Atypical laterality of resting gamma oscillations in autism spectrum disorders. J Autism Dev Disord. (2015) 45:292–7. 10.1007/s10803-013-1842-7 23624928 PMC6102725

[43] Sase T Kitajo K. The metastable brain associated with autistic-like traits of typically developing individuals. PLoS Comput Biol. (2021) 17:e1008929. 10.1371/journal.pcbi.1008929 33861737 PMC8081345

[44] Watanabe T Rees G. Brain network dynamics in high-functioning individuals with autism. Nat Commun. (2017) 8:16048. 10.1038/ncomms16048 28677689 PMC5504272

